# Coexistence between human and wildlife: the nature, causes and mitigations of human wildlife conflict around Bale Mountains National Park, Southeast Ethiopia

**DOI:** 10.1186/s12898-020-00319-1

**Published:** 2020-09-14

**Authors:** Sefi Mekonen

**Affiliations:** Department of Biology, College of Natural and Computational Science, Debre Berhan University, Debre Berhan, Ethiopia

**Keywords:** Biodiversity, Conservation, Human–wildlife interactions, Management, Protected area

## Abstract

Human–wildlife conflict occurs when the needs and behavior of wildlife impact negatively on humans or when humans negatively affect the needs of wildlife. To explore the nature, causes and mitigations of human wildlife conflict, the coexistence between human and wildlife assessment was conducted around Bale Mountains National Park. Data were collected by means of household questionnaires, focus group discussion, interview, field observation and secondary sources. The nature and extent of human wildlife conflict in the study area were profoundly impacted humans, wild animal and the environment through crop damage, habitat disturbance and destruction, livestock predation, and killing of wildlife and human. The major causes of conflict manifested that agricultural expansion (30%), human settlement (24%), overgrazing by livestock (14%), deforestation (18%), illegal grass collection (10%) and poaching (4%). To defend crop raider, farmers have been practiced crop guarding (34%), live fencing (26%), scarecrow (22%), chasing (14%), and smoking (5%). However, fencing (38%), chasing (30%), scarecrow (24%) and guarding (8%) were controlling techniques to defend livestock predator animals. As emphasized in this study, human–wildlife conflicts are negative impacts on both human and wildlife. Accordingly, possible mitigate possibilities for peaceful co-existence between human and wildlife should be create awareness and training to the local communities, identifying clear border between the closure area and the land owned by the residents, formulate rules and regulation for performed local communities, equal benefit sharing of the local communities and reduction of human settlement encroachment into the national park range. Generally, researcher recommended that stakeholders and concerned bodies should be creating awareness to local community for the use of wildlife and human–wildlife conflict mitigation strategies.

## Introduction

Human–wildlife conflict (HWC) occurs when the needs and behavior of wildlife impact negatively on humans or when humans negatively affect the needs of wildlife. These conflicts may result when wildlife damage crops, threaten, kill or injure people and domestic animals [33]. These are as critical problems created by the growing rural population in and around wildlife habitats [34]. Human–wildlife conflict incidents are widespread but not evenly distribute because they are dependent on the proximity of wildlife. In addition, different species cause different types of damage at different times of the year. The damage caused has variable effects on the livelihood of households depending on their level of livelihood security at the time of the incident [27]. One major cause of human–wildlife conflict is increasing human population adjacent to wildlife habitats. As human population increases and the demand for resources grow, the frequency and intensity of such conflicts increases [29]. This can be manifested by increasing encroachment to wildlife habitats. As a result, the populations of those species which are unable to adapt to altered habitats may invade the marginal habitats or decline in number [26, 29]. Human wildlife conflicts undermine human welfare, health, safety and have economic and social cost [31].

Because HWC is a reciprocal process, humans and animals are negatively affected by the conflict, and it is one of the most complex and urgent issues facing wildlife management and conservation [10], especially outside PAs [38]. Scholars are seeking ways to refocus policy-relevant conflict research on finding pathways toward human–wildlife coexistence [23] and coadaptation [2]. According to König et al. [18], literature on HWCs, interaction, and coexistence has grown exponentially from 2000 to 2019, and work on conflict outpaces work on interactions and coexistence threefold. This may be because scholarship on human–wildlife interactions has focused mainly on conflict (i.e., negative outcomes for people, wildlife, or both) [3] or because new ways of thinking about these interactions now include a paradigm of coexistence [18]. Coexistence is defined as a dynamic but sustainable state in which humans and wildlife co-adapt to living in shared landscapes, where human interactions with wildlife are governed by effective institutions that ensure long-term wildlife population persistence, social legitimacy, and tolerable levels of risk [2, 18]. The focus on human–wildlife conflict has often been a constraint to wildlife conservation, as practitioners have centered their attention on reducing negative interactions, rather than on increasing positive relations between humans and wildlife. To work toward solutions that maximize conservation success, it is necessary to include positive interactions, coexistence, and attitudes of tolerance toward wildlife [11].

Human wildlife conflict is a well-known phenomenon throughout sub Saharan Africa [34]. Reducing human–wildlife conflict is also an urgent conservation priority and key to coexistence between humans and animals in this region [37]. There are many human wildlife conflicts in Ethiopian protected areas which need solutions, but there is no enough empirical study done on issues pertaining to human wildlife conflict. Local communities do not enough benefit from wildlife resources and alienated from wildlife related economic enterprises. Like other parks or protected areas in Ethiopia, Bale Mountains National Park (BMNP) is highly influenced by the human activities living in and around the Park. Due to this, local peoples around the park and the wildlife are being affected negatively as the result this interaction. From this perspective, it is imperative to assess the human–wildlife conflict that occurs with local communities living adjacent to BMNP. There was no detail investigation made to identify the cause of the conflict and its adverse consequences.

Identify potential ways to reduce or prevent conflicts for the better wellbeing of both people and wildlife is the main objective of conducting research on human–wildlife conflict [21]. A prerequisite for finding effective solutions is understanding the details, mechanisms, and nature of conflict [21]. Therefore, this particular study aims to investigate the nature, extent, roots and mitigations of human wildlife conflict in and around the park. Moreover, this study was serving as ideal or standard information for the coexistence between humans and wildlife. Local community, researchers and other organizations are benefited from the study by getting how to conserved or managed the wildlife in additional to ensure positive coexistence between people and wild animal in the interest of human and environmental wellbeing.

## Materials and methods

### Description of the study area

The study area Bale Mountains National Park is located southeast of Ethiopia about 400 km by road from Addis Ababa, between 6^o^ 29′ and 7^o^ 10^o^ North and 39^o^ 28′ and 39^o^ 58^o^ East. It covers an area of 247,000 hectare of land with an altitudinal range from 1500 to 4377 m asl [8]. It was first proposed in the late 1960 s to protect Afroalpine habitat and populations of the rare, endemic and species of the mountain nyala (*Tragelaphus buxtoni*), the giant molerat (*Trachyoryctes macrocephalus*) and the Ethiopian wolf (*Canis simensis*) [16], [1]. Even if its establishment was for this Bale Mountains is one of the most important areas of the world for its number of threatened Ethiopian endemics in all taxa 26% of Ethiopia’s endemic species. Of the area’s recorded birds, 6% are Ethiopian endemics. In addition there are several rare and endemic amphibian species found only in Bale as well as 1321 species of flowering plants with 163 endemic (23 to Bale alone) to Ethiopia [16].

The Park, as part of the highlands of eastern Africa encompasses a variety of habitats that supports a diversity of wildlife species. The habitat types include grassland, woodland, heather moorland and Afro-alpine vegetation [14, 15, 24]. Bale Mountain National Park is undoubtedly one of the most unique areas on earth, with the largest piece of Afro-alpine habitats with the second largest moist tropical forest and the only cloud forest in Ethiopia [16]. And it is an Important Bird Area of immense importance comprising more than 256 species of birds with seven endemics from the afro tropical highland biome species which represents 80% of the species making the area the richest site for this biome assemblage [1, 8, 16].

### Data collection methods

The data were collected through primary and secondary methods. Primary data was collected from household questionnaires, interview and field observation. Household questionnaires were implemented to gather the data related to assess HWC cause, the nature of conflict and management and mitigation strategies to reduce conflict in the area. The study was based on mainly park office and sample kebele household cross sectional survey using pre-test structure questioner organizing in logical order of presentation. Key informant interviews were conducted with selected informants who are depending on park resources. Interview was held with park scout, park manager and local community to explore the nature of HWC and human and wildlife coexistences. In focus group discussions, the researcher is just a facilitator and the respondents provide information. Focus groups therefore, provided an opportunity for the researcher to interact with the local community and gain relevant information about their knowledge, opinions, and attitudes regarding human–wildlife conflicts and to determine effective HWC management and control methods used by local people. Field observation was mainly used to confirm the respondent’s responses, so that accurate and reliable information would be collected during filed observation. The observation was carried out in three purposive selected kebeles (Dinsho Kebele 01, Goba and Rira kebele). To make the research more reliable and to obtain an objective data which is got from primary data was supported by the secondary one. Secondary data collection sources are data obtained from books, internet searches, libraries, journal, progress reports, Park office and articles.

### Sampling size and sampling technique

It is obvious that Bale Mountains National Park is surrounded by five Districts such as: Adaba (west), Dinsho (north), Goba (northeast), Mana-Angetu (south) and Berbere (east). However, the extent of exposure of local people and their agricultural area to wildlife is not the same throughout the five Districts rather it greatly differs from one to another. Therefore, two Districts (Dinsho and Goba Woreda) were selected using systematic random sampling technique through careful identifications in which those which have high extent of exposure with the park boundaries. In addition, random sampling technique was employed to identify sample households. In this heads of households were randomly selected from sample kebeles/villages of the two Districts which were selected using systematic random sampling after the completion of preliminary survey which is helpful to identify specific villages which are highly affected as a result of the conflict with wildlife. 5% of the total households from each sample village were selected randomly.

The sampling size of the study was determined based on formula adapted from Israel (1962) as follows.

$${\text{n}} = {\text{N}}/1 + {\text{N }}\left( {\text{e}} \right)^{2}$$

where; N = the total population; n = the required sample size; e = the precision level which is = (± 10%), where confidence interval is 90% at p = + 10 (maximum variability) which is = (± 10%) n = 1850/1+1850(0.1)^2^ = 95.

Accordingly, from the total (1850) population of three villages, a total of 95 respondents were selected and the questionnaire was transferred purposefully. The respondents were selected purposively based on their ability, awareness, adjacent to an area and knowledge contributes to the overall research objectives.

### Data analysis

The data was analyzed by using simple descriptive (qualitative) method and quantitative (numerical) method. The study was interpreting the data based on the survey questionnaire, interview and filed observation. The data was analyzed by using simple descriptive statistics such as mean percentage and the data was present on tables, charts, picture and percentage also further represented by using graphs and other diagram in order to analyses more information about our research study.

## Results

### Nature and extent of human wildlife conflict

The nature and extent of human wildlife conflict in and around Bale Mountains National Park have profoundly impacted humans, wild animal and the environment in many ways through crop damage, habitat disturbance and destruction, livestock depredation, killing of wildlife and human and the like. As a result, local communities disliked wildlife inhabiting in and around their surroundings. This has a great negative impact in conservation of the wildlife.

### Crop damage

**Fig. 1:**
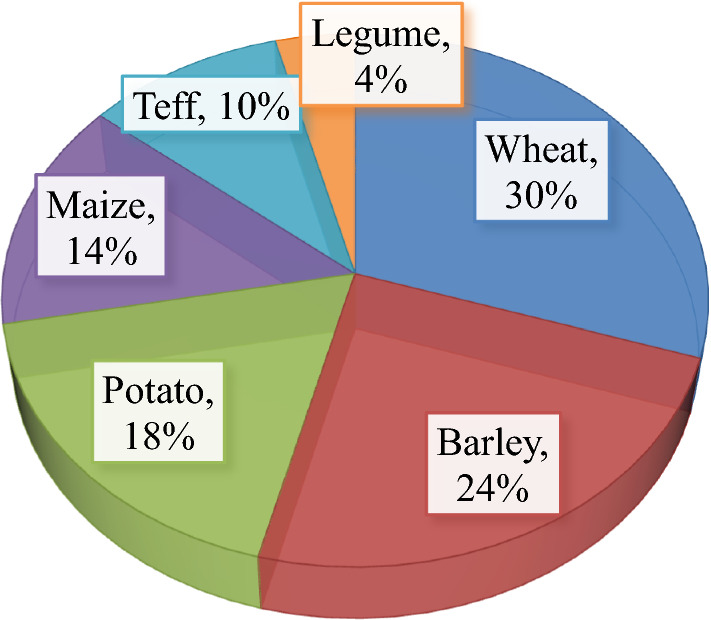
Rank of vulnerable crops in the order of destruction by crop raider

The result showed that not all crops were equally affected by crop raiders (herbivore wild animals) in the studies area. Olive baboon (*Papio anubis*), warthog (*Phacochoerus aethiopicus*), common mole rat (*Tachyoryctus splendens*), porcupine (*Hystrix cristata*), grey duiker (*Sylvicapra grimmia*), mountain nyala (*Tragelaphus buxtoni*) and bohor reedbuck (*Redunca redunca*) were mentioned as important crop raiders. According to farmers, Olive baboon (*Papio anubis*) was the most commonly reported crop raiders which cause more damage and ranked first followed by warthog (*Phacochoerus aethiopicus*). They damage crops early in the morning and evening when people are absent near farmlands. While, respondents were putted porcupine (*Hystrix cristata*) are as third crop raiders followed by bohor reedbuck (*Redunca redunca*). Human and wildlife have been in conflict because farming crops generally offer a rich food source for wildlife as well as for people. Large wild herbivores compete for fallow resources with livestock and can act as reservoirs of livestock diseases. The respondents claimed that Wheat (30%) and barley (24%) was the most vulnerable crop to raiders’. Whereas respondents reported that potato, Maize, Teff and legume are damaged by wild animals on rank 18%, 14%, 10% and 4%, respectively (Fig. 1).

### Habitat disturbance

Habitat disturbance is destruction of the home of the wild animals. Humans kill or chase wild animals by digging, cutting, sealing by stones and smoking their natural habitat. This method is a main cause to decrease or to extinct of wild animals. The major components of habitat disturbance in the study area were settlement in and around the national park, over grazing by livestock, frequent fire and bush encroachment, tree cutting for charcoal, sale and construction of huts. Tree cutting was mainly associated with new settlement, which resulted deterioration of the remaining vegetation cover of the area. This minimizes the feeding ground, nesting and mating site of the wild animals so you have to be happened conflict between human and wild animal.

### Livestock depredation

**Fig. 2:**
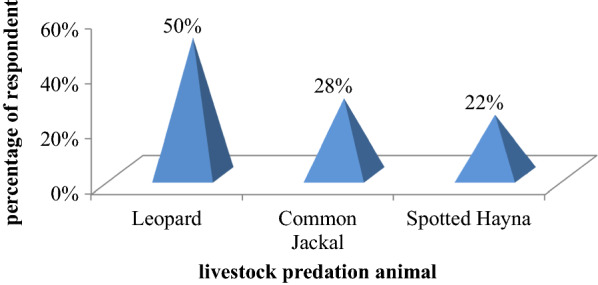
Major livestock depredation wild animal in the study area

According to the respondents a total of three (namely, leopard (50%), Common Jackal (28%) and spotted hyenas (22%)) common problematic wild animals were reported in terms of livestock depredation from the villages although their effect is differing from village to village (Fig. 2). Leopards were reported to attack cattle, donkeys, goats, sheep and domestic dog in the study area. Common jackals are attack sheep; goat and spotted hyena caused the most pronounced problems and the local communities’ loss their oxen, cows, donkeys, mules, domestic dog and horses. Carnivores are attacking domestic livestock due to declining number of herbivorous in the wild due to prolonged droughts and habitat degradation.

### Killings of wildlife

Because of lack of compensated for crop losses, and domestic animal killing or loss the local communities are more suffered by wild animals and then they straggled to kill wild animals. This study was showed that Crop-raiding undermines food security and intolerance of wildlife within neighboring human communities in the study area. The inability to mitigate crop-raiding and absence of composition for crop losses lead to killing of animals.

### Root causes of human–wildlife conflicts

**Fig. 3:**
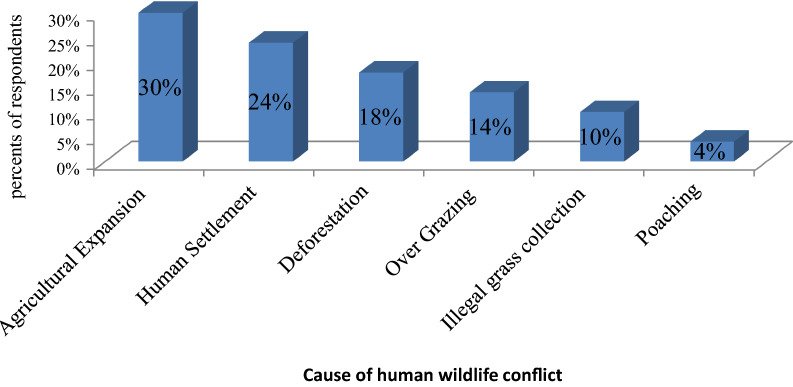
Cause for Human wildlife conflict in and around the study area

**Fig. 4:**
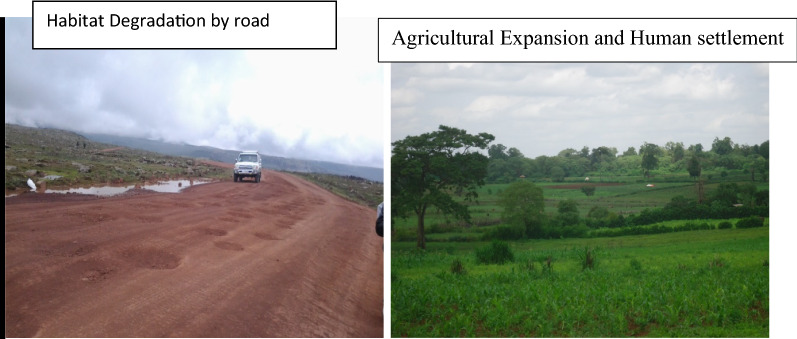
Habitat Degradation, Agricultural Expansion and human settlement

According to the respondents and field observation, the main root causes of human wildlife conflict in the study area were: agricultural expansion (30%), human settlement (24%), overgrazing by livestock (14%), deforestation (18%), illegal grass collection (10%) and poaching (4%) (Figs. 3 and 4).

**Fig. 5:**
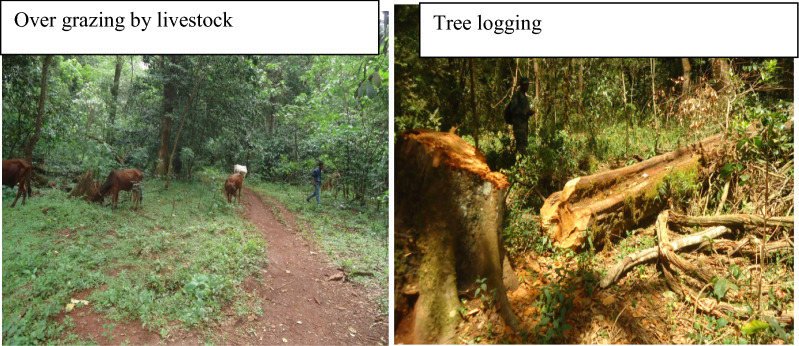
Over grazing and tree logging by livestock

Deforestation is another major cause of human wildlife conflict in the study area mainly caused by cutting of trees for expansion of farm land, fire wood collection and livestock grazing send fire for the purpose of charcoal production (Fig. 5). Over grazing also was another major cause of human wildlife conflict the in the study area. This cause was due to the local communities were farming and livestock production are the main activities.

### Minimizing and mitigations of human wildlife conflict

According to the respondents, different methods are used by farmers to defend crop raider from their crop include crop guarding (34%), live fencing (26%), and scarecrow (22%), chasing (14%), whereas 5% was used smoking to repeal the crop raiders from their crop mostly in the night time which was the lowest method (Table 1).

**Table 1:** Controlling techniques of crop raiding animal

No.	Controlling Techniques	No. of Respondents	Percent (%)	Rank
1	Guarding	32	34	1
2	Live fencing	25	26	2
3	Scarecrow	21	22	3
4	Chasing	13	14	4
5	Smoking	4	4	5

**Fig. 6:**
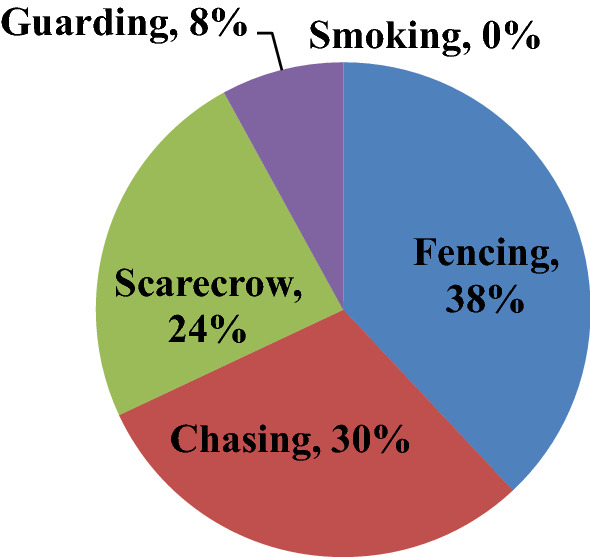
Traditional controlling techniques of livestock predation animal

As below Fig. 6 showed, the local community used different controlling techniques to defend livestock predator animals, such as fencing (38%), chasing (30%), scarecrow (24%), guarding (8%), and smoking (0%) based on respondents rank. These traditional controlling techniques of the most effective methods are fencing and chasing, the second most effective methods are scarecrow and guarding (especially common jackal) and the least effective traditional controlling techniques are smoking.

## Discussion

Wildlife damage to agricultural crops is a serious concern affecting much of the world today [30]. Primates are one of the most frequently cited crop pests [13], so primates and humans are always in potential conflict over crops. This conflict is particularly interesting in that it arises from a positive desire to contact monkeys and then people discover that the contact poses risks from bites, theft of non-provisioned food or more general health issues such as exposure to simian viruses [6]. The result was agreed with finding of [36] who reported that wheat (ripe and dried) was the most frequently eaten crop by crop raiding in West Africa. Study conducted in Rwandan Forest Fragment indicated that maize, potato, beans, cabbage, sweet potato and tomato were raided by wild animals [12].

A research conducted in and around the study area showed that Olive baboon, warthog, common mole rat and bohor reedbuck (Redunca redunca), were identified as destructive animals, mainly feeding commonly on wheat, barley, potato, maize, teff and legume. Similar finding with the current study was observed in Filinga Range of Gashaka Gumti National Park of Nigeria. Monkeys, Baboons, Birds and Rodents were listed among wild animals that attack crops including Maize, Cassava, Rice and Banana [7].

Hence, common jackal and leopards could easily penetrate the fences and drag out the sheep and goat and any other animals. So, most of the predation by leopard happened during Both Night and Day Time but common jackal happened in day time and Spotted Hyena during happened in the night time within the settlement. This result is the same as with [25] that reported Leopard, Spotted Hyena and Common jackal were the major predators for domestic animals in and around Semen Mountains National park of Ethiopia. They were responsible for loss of Sheep, Goats, Oxen, Cows, Donkeys and Mules. Eight problematic wild animals in terms of domestic animal loss were identified in Chebera Churchura National Park southwestern part of Ethiopia [5]. Among those hazardous wild animals three of them i.e. Leopard, Jackal, and hyena were same with the present finding.

Reduction in the availability of natural prey/food sources leads to wild animals seeking alternate sources. Alternately, new resources created by humans draw wildlife resulting in conflict [39]. Byproducts of human existence offer un-natural opportunity for wildlife in the form of food and sheltered interference and potentially destructive threat for both man and animals. Competition for food resources also occurs when humans attempt to harvest natural resources such as fish and grassland pasture. Another cause of conflict comes from conservation biased toward flagship or game species that often threatens other species of concern [20].

According to the respondent’s response (10%), the local people cut grass illegally to feed their cattle, sell in the market and for thatching houses. This might cause scarcity of grass for herbivores and disturb the natural behavior of wildlife in the Park. Like any other Park in Ethiopia, local people exploit the resource from BMNP as well. Forest exploitation inside the Park and traditional farming activities close to the Park might cause strong impacts on the wildlife. Wild animals are highly restricted in some parts of the Park because of human and livestock encroachment.

Therefore the researcher was concluded that Agricultural Expansion (30%) and human settlement (24%) are the major causes of human wildlife conflict in and around the study area (Figs. 3, 4, and 5), while poaching (4%) are the lowest cause of HWC. Recently there was agricultural practice and human settlement inside and outside the park. Similar sources for Human wildlife were reported from Tsavo Conservation Area, Kenya. Agricultural Expansion, human settlement, deforestation, illegal grass collection, poaching was reported as the main causes of Human wildlife conflicts [22]. Different causes for human wildlife conflict were reported from different parts of Africa. For instance, animal death, loss of human life, crop damage, and damage to property, injuries to people and wildlife, encroachment of forest areas for agriculture, developmental activities, and livestock grazing are some key reasons for increment of the conflict in countries such as Kenya, Namibia, Mozambique, Zambia and Nigeria [19].

Many traditional repelling techniques are fairly effective if formalized, but are labor intensive. But where an animal can be repelled adequately using conventional methods it seems in appropriate, and certainly not particularly cost effective to try to introduce more expensive techniques requiring greater technological input or backup [4]. Another approach that has been used successfully to manage Human wildlife conflict involves changing the perceptions of people experiencing the damage, thus, increasing their willingness to tolerate damage [35]. Agricultural producers already are receptive to this argument and appreciate the wildlife on their farms to enhance wildlife habitat and their tolerance for some wildlife damage. This tolerance can be enhanced by providing economic incentives [25].

There was percentage difference between respondents using the different traditional methods in which of the respondents were used to defend their crop from crop raiders. This result agrees with the finding of [17] who founds that guarding and live fencing away of animals was ranked first and second in protecting crop raiders from crops. According to [13], the most viable options to reduce crop loss were increasing vigilance by farmers. This has been shown to make a considerable difference in the amount of crops lost, increasing farmer tolerance for a pest species and lost crops and increasing the ability of farmers to repel crop raiders using existing local methods. This has a number of obvious benefits, if these methods do not make a considerable impact on crop loss, and larger impact interventions such as electric fencing, lethal control of pest animals or moving farmers from the conflict zone can be considered [35].

Selection of the different strategies depends on the type of species, behavior of species and size of species. These results were similar to reported from Kenya Nyeri district [28]. The most effective strategy of the local communities used in preventing crop damage was guarding (34%), which is time consuming [7]. Similarly, the communities in the present study reported that permanent Guarding by adults is the most effective strategy to control both crop and livestock from wildlife when asked the most effective deter strategy among practiced by the local people. Active guarding by famers and members of their families was found to be the sole mode of protection from crop raiding [12].

No single management strategy can prevent all crop raiding and the goal of management should not only to be reducing the levels of crop raiding but also to raise the tolerance level of crop raiding by lessening its impact to farmers [33]. No solution will work without site-specific knowledge of what is possible, practical, or acceptable in any particular area. Unfortunately, human–wildlife conflict situations are often complex so are unlikely to be resolved quickly and cannot be solved solely by technical means. Human wildlife conflict can be managed through a variety of approaches. Prevention strategies endeavor to avoid the conflict occurring in the first place and take action towards addressing its root causes [13]. The main difference between the options is the moment at which the measure is implemented. By definition, management techniques are only cost-effective if the cost of implementing the technique is less than the value of the damage, taking into account the fact that a short period of active management may have a continued effect, by instating longer-term protection of crops or herds [9]. The various management possibilities are presented according to the characteristics of conflict whether they relate to humans, production, animals and the environment, rather than according to their ability to prevent or mitigate damage [20].

According to Hill et al. [13], conflict resolution/management methods have the following possible goals: reducing the amount of crop losses to wildlife; improving local people’s attitudes and perceptions towards protected area and its wildlife; helping affected farmers to improve agricultural production; increasing the amount of crops being harvested locally. Through improved local yields and reducing levels of poaching. Those wise it is very important that farmers be involved in the process of developing new solutions from the beginning [35]. Not only does this foster a sense of commitment and involvement amongst them, but it is also vital that they be involved from the beginning. Because they understand how the situation affects them and what kinds of intervention are likely to be acceptable and feasible with in the local culture, providing there is adequate representation from the different types of stakeholder involved [32].

The present study showed that human wildlife conflict is apparent in the study area. The conflict becomes the main causes to the continued survival of wild animal species in the area. Not only causes for wild animals but also the conflict causes high impact in economic loss of the people in and around the study area. Therefore, human–wildlife conflicts are negative impacts on both human and wildlife as highlighted in this study. It is also a serious obstacle to wildlife conservationists. Based on these reasons, mitigation strategies are very essential to reduce the cause and impact of HWC. Accordingly, possible mitigate possibilities for peaceful co-existence between human and wildlife are presented as follows:- Create awareness and organize training program to the local communities, identifying clear border between the closure area and the land owned by the residents, rules and regulations of the park, translocate the problematic animal to another area, equal benefit sharing of the local communities, to reduce or minimize agricultural practice inside and outside the national park, reduce deforestation by formulate rules and regulation for performed local communities, relocate agricultural activity out of the national park range, zoning or change the location of crop fields, Reduction of human settlement encroachment into the national park range.

## Conclusions

The result of the present study has clearly shown that there was a strong conflict between human and wildlife living in and around the study area. The cause of human wildlife conflict was human settlement, agricultural expansion, illegal grass collection, over grazing by livestock and deforestation in national park. As a result, local communities disliked wildlife inhabiting in and around their surroundings. This has a great negative impact in conservation of the wildlife. The main effects for the presence of strong human wildlife conflict in the study area include crop damage, livestock depredation, killing of wildlife and habitat disturbance. Therefore, determination of possible solutions to mitigate Human wildlife conflict in the study area is mandatory for peaceful coexistence of human and wildlife.

Based on the obtained results of the present study, the following points are recommended in the study area:

- Farmers should cooperatively keep their farm against crop raiders to minimize crop loss by using most effective method in an area.
- The park authority should provide compensation for wildlife induced damage in and around the park.
- Palatable and nutritive crops should not be grown near the park edge.
- The concerning body should work hard to increase the awareness of the local people about the importance of wildlife conservation.
- The park authority should provide fence or other method that used to protect crops, peoples and livestock from threat.
- Stakeholders should reduce human settlements around the forest, expansion of farmland and cattle grazing in and around the National Park.
- To reduce the dependency of the local people in and around the national park, it is better to encourage the local people to plant trees for their various types of utilization.
- Further investigation must be conducted to identify alternative crops that can be rejected by crop raiders in the area.

## Data Availability

The data used and analyzed during the current study is available from the corresponding author on a reasonable request, without disclosure of the interviewees.

## Abbreviations

IBC: Institute of Biodiversity Conservation
BMNP: Bale Mountains National Park
HWC: Human wildlife conflict
